# Ferroptosis Signatures in Diabetic Cardiomyopathy: Multi-Omics Discovery and Validation of ACOT1 and TXNIP

**DOI:** 10.3390/cimb48090923

**Published:** 2026-09-09

**Authors:** Feng Zhou, Jia-Bin Zhou, Ling Zhang, Yi-Qing Yan, Dan Wu, Tian-Peng Wei, Zhen-Ye Zhang, Huan-Huan Liu, Jun-Xian Shen, Ying Liu, Ling-Ling Qian, Ru-Xing Wang

**Affiliations:** Department of Cardiology, Wuxi People’s Hospital Affiliated to Nanjing Medical University, Wuxi Medical Center, Nanjing Medical University, Wuxi 214023, China; njmu2024zf@163.com (F.Z.); zhoujiabin2021@163.com (J.-B.Z.); lingzhang07@163.com (L.Z.); lightsmith0325@163.com (Y.-Q.Y.); wudan787999@163.com (D.W.); wech612@163.com (T.-P.W.); zhangzhenye@njmu.edu.cn (Z.-Y.Z.); hhliu1224@163.com (H.-H.L.); junxianshen326@163.com (J.-X.S.); stu_liuying@163.com (Y.L.); qianlingling@njmu.edu.cn (L.-L.Q.)

**Keywords:** diabetic cardiomyopathy, ferroptosis, bioinformatics, ACOT1, TXNIP

## Abstract

**Background:** Diabetic cardiomyopathy (DCM) is a serious cardiovascular complication specific to diabetes mellitus, with rising global prevalence. Ferroptosis, an iron-dependent form of regulated cell death driven by lethal lipid peroxidation, has been implicated in the pathogenesis of DCM. However, the key regulatory genes remain poorly characterized. This study aimed to identify and validate ferroptosis-related signature genes in DCM. **Methods:** Three murine transcriptomic datasets (GSE123975, GSE155377, and GSE210611) were retrieved from GEO and merged after batch correction. Differentially expressed genes were intersected with weighted gene co-expression network analysis disease-associated module genes and FerrDb ferroptosis annotations to define the ferroptosis-related differentially expressed gene candidate pool. LASSO regression and random forest selection then prioritized hub genes, defined operationally as candidates consistently prioritized by both machine-learning algorithms rather than by network-topological centrality. Classification performance was evaluated by ROC analysis and validated in two independent cohorts (GSE161931 and GSE274500). mMCPcounter estimated immune and stromal infiltration. ScRNA-seq (GSE290095) and spatial transcriptomic (GSE290094) profiling characterized cellular distribution, predicted cardiomyocyte network perturbations and tissue-level expression patterns. High-fat diet/streptozotocin (HFD/STZ)-induced DCM rat models provided experimental validation. **Results:** *Acot1* and *Txnip* were identified as hub genes, with strong discriminatory performance in the discovery cohort (AUC = 1.000 and 0.988; in-sample estimates, *n* = 26) and independent external validation (AUC = 0.951 and 0.988). Immune profiling linked both genes inversely with vessel scores, and *Txnip* was also linked with eosinophils. Single-cell analysis localized *Acot1* enrichment to cardiomyocytes and endothelial cells, while *Txnip* was broadly expressed across multiple cell types, with elevated levels in DCM. In silico knockout analysis predicted distinct cardiomyocyte network perturbation profiles for *Acot1* and *Txnip*, and spatial transcriptomics revealed modest but disease-specific spatial associations between hub gene expression and ferroptosis driver scores (*Acot1*: rho = 0.123; *Txnip*: rho = 0.154). Both genes were significantly upregulated at mRNA and protein levels in HFD/STZ-induced DCM rats, with concurrent GPX4 depletion, ACSL4 accumulation, and FTH1 reduction consistent with ferroptosis activation. **Conclusions:** This study identifies *Acot1* and *Txnip* as ferroptosis-related molecular signatures in DCM and provides multistep prioritization and validation spanning bulk transcriptomics, single-cell and spatial transcriptomics, and in vivo experimental verification, offering potential targets for ferroptosis-targeted therapeutic intervention.

## 1. Introduction

Diabetes mellitus represents one of the most pressing global health challenges of the twenty-first century. It is estimated that the worldwide prevalence of diabetes will be approximately 12.96% by 2050, corresponding to an estimated 853 million affected individuals [1]. Cardiovascular complications constitute the leading cause of morbidity and mortality among patients with diabetes [2]. Among these, diabetic cardiomyopathy (DCM) is a distinct clinical entity defined as myocardial dysfunction occurring independently of coronary artery disease, uncontrolled hypertension, or other identifiable cardiac pathologies [3]. DCM follows an insidious clinical trajectory, progressing from early diastolic impairment and myocardial hypertrophy to overt systolic dysfunction and clinical heart failure [4,5].

The pathogenesis of DCM is multifactorial. Chronic hyperglycemia and insulin resistance act as upstream drivers, triggering a cascade of downstream events including enhanced oxidative stress, mitochondrial dysfunction, endoplasmic reticulum stress, chronic low-grade inflammation, and disrupted calcium homeostasis [6,7]. These interconnected processes converge to promote cardiomyocyte injury, interstitial fibrosis, and progressive cardiac remodeling [8,9]. Among the various forms of regulated cell death implicated in this process, ferroptosis, an iron-dependent cell death mechanism driven by lethal lipid peroxide accumulation and redox homeostasis collapse, has attracted increasing attention [10,11]. In the diabetic myocardium, iron overload, excessive reactive oxygen species (ROS) generation, and impaired glutathione peroxidase 4 (GPX4) function collectively create a ferroptosis-permissive microenvironment [12,13]. Pharmacological inhibition of ferroptosis has been shown to attenuate diabetes-related myocardial damage [9]. However, the regulatory genes and molecular networks governing ferroptotic susceptibility in DCM remain to be systematically defined.

The integration of high-throughput transcriptomic profiling with machine learning has become a standard approach for prioritizing candidate gene signatures in complex diseases [14,15]. In particular, weighted gene co-expression network analysis (WGCNA) combined with feature selection algorithms such as LASSO and random forest (RF) enables the identification of disease-associated genes from high-dimensional data with improved robustness [16,17]. When coupled with ferroptosis-specific gene annotations and multi-resolution spatial validation, such a framework may offer additional specificity for dissecting ferroptosis-related molecular alterations in DCM.

Recent bioinformatics studies have begun to apply integrative approaches to DCM, identifying candidate genes involved in metabolism, immune regulation, and endoplasmic reticulum stress [18,19,20]. Building on these efforts, we coupled ferroptosis-directed candidate filtering with validation across multiple resolutions, including cross-platform external bulk cohorts, single-cell and spatial transcriptomics, and an in vivo DCM model, to prioritize and corroborate ferroptosis-related signatures. This layered design moves beyond single-dataset gene lists toward signatures whose disease association is reproducible across platforms and supported at tissue resolution.

**Figure 1:**
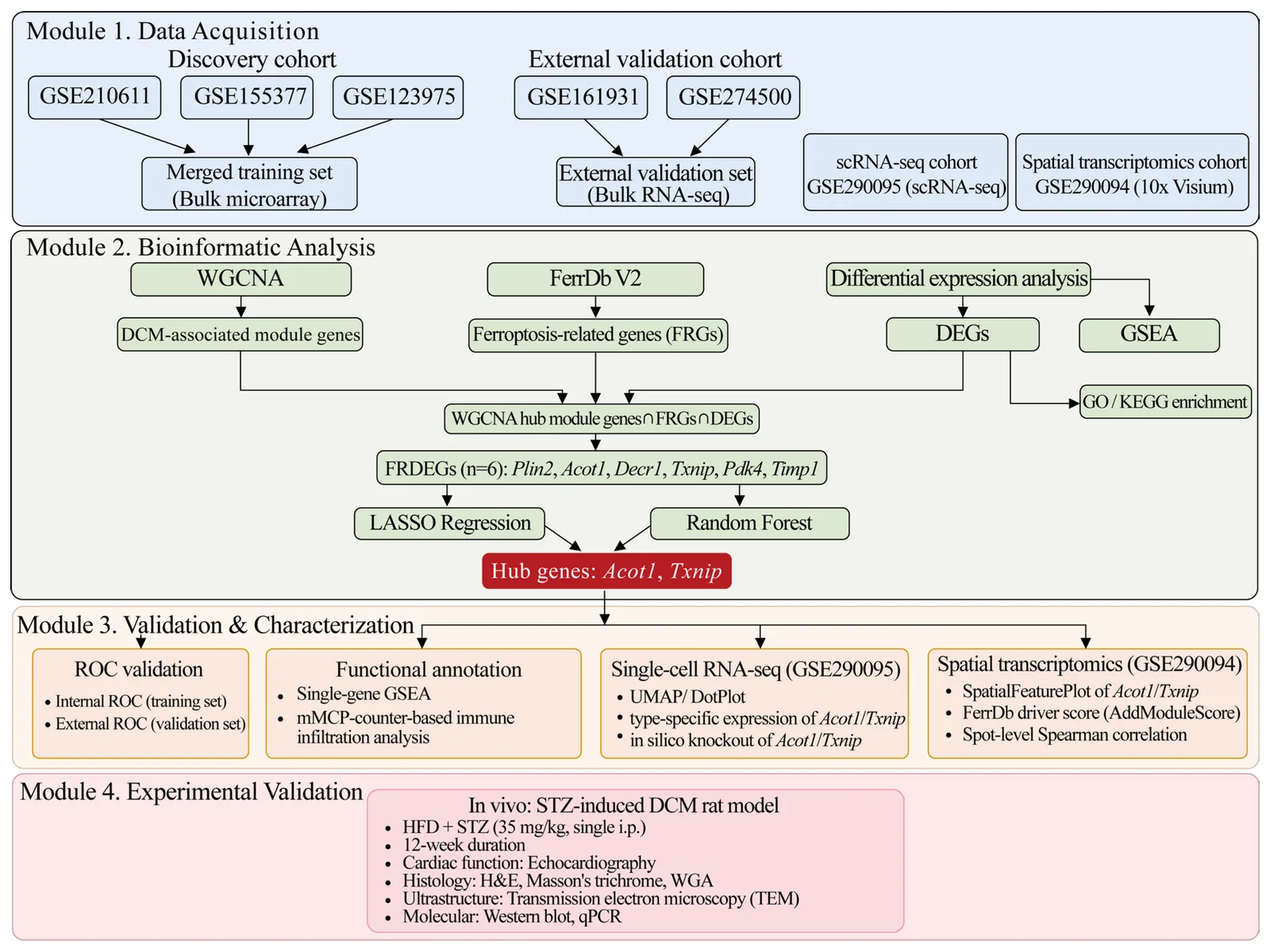
Workflow of the study. The study consisted of four modules: data acquisition, bioinformatic analysis, validation and characterization, and experimental validation. Hub genes (*Acot1* and *Txnip*) were identified and validated across multiple cohorts and in an STZ-induced DCM rat model. DCM—diabetic cardiomyopathy; STZ—streptozotocin.

In the present study, we integrated three murine transcriptomic datasets and employed WGCNA combined with dual machine learning algorithms to identify ferroptosis-related signature genes in DCM. The identified hub genes were validated through independent external cohort analysis, immune infiltration profiling, single-cell and spatial transcriptomic characterization, and experimental verification in vivo. This study aims to provide molecular insights into the ferroptosis-associated transcriptomic landscape of DCM and to identify candidate targets for further mechanistic and therapeutic investigation. The overall workflow of this study is shown in Figure 1.

## 2. Materials and Methods

### 2.1. Data Acquisition and Preprocessing

Three murine DCM transcriptomic datasets were retrieved from the GEO: GSE123975 (6 DCM, 6 Con; GPL10787), GSE155377 (4 DCM, 4 Con; GPL23038), and GSE210611 (3 DCM, 3 Con; GPL21810), all microarray-based. Probe-level intensities were mapped to gene symbols using platform annotation files, and multiple probes mapping to the same gene were collapsed by arithmetic mean. The datasets were merged into a unified expression matrix comprising 26 samples (13 DCM, 13 Con). Inter-study batch effects were corrected using the ComBat algorithm (*sva* R package), using a design matrix that encoded disease status while preserving biological variation [21].

### 2.2. Identification of Differentially Expressed Genes

Differentially expressed genes (DEGs) between DCM and control groups were identified using the *limma* R package [22,23]. A linear model was fitted with empirical Bayes variance moderation, and genes with *p* < 0.05 and |log_2_FC| > 0.5 were designated as nominally significant DEGs. DEGs were visualized using a volcano plot and a heatmap of the top 25 upregulated and top 25 downregulated genes. As a sensitivity analysis, DEGs were additionally defined using a BH-adjusted *p* < 0.05 threshold.

### 2.3. Functional Enrichment Analysis

Gene Ontology (GO; biological process, cellular component, molecular function) and KEGG over-representation analyses were performed on DEGs. Gene Set Enrichment Analysis (GSEA) was performed on the gene list ranked by moderated t-statistics using the *clusterProfiler* R package with KEGG annotations [24]. Enrichment significance was assessed with Benjamini–Hochberg correction (adjusted *p* < 0.05). A custom ferroptosis gene set curated from FerrDb V2 was additionally subjected to GSEA to evaluate enrichment of ferroptosis-related pathways [25].

### 2.4. Weighted Gene Co-Expression Network Analysis

WGCNA was implemented using the *WGCNA* R package [19,26]. Genes in the batch-corrected matrix were filtered to the top 50% by variance. A soft-thresholding power of β = 6 was selected based on the scale-free topology criterion (signed R^2^ ≥ 0.90), indicating approximate scale-free topology. A topological overlap matrix (TOM) was computed and hierarchically clustered (average linkage); modules were identified via dynamic tree cutting (deepSplit = 1, minimum module size = 50) and merged at an eigengene dissimilarity cutoff of 0.25. Module eigengenes were correlated with binary disease status (DCM/Con), and modules with |cor| > 0.7 and *p* < 0.05 were retained for downstream analyses.

### 2.5. Definition and Machine-Learning Selection of Ferroptosis-Related Hub Genes

Ferroptosis-related differentially expressed genes (FRDEGs) were defined as genes at the intersection of DEGs, WGCNA module genes, and FerrDb ferroptosis genes, visualized by Venn diagram. Hypergeometric tests were used to assess whether the observed set overlaps exceeded chance expectation, with the WGCNA input gene set (top 50% most variable genes, N = 9071) as the eligible background. Two machine learning approaches were applied for hub gene selection. LASSO logistic regression (*glmnet* R package) was performed with cross-validation to identify genes retaining non-zero coefficients at the optimal penalty parameter. RF classification (*randomForest* R package) ranked candidate genes by mean decrease in Gini impurity, with the number of trees optimized by minimizing out-of-bag error. Genes consistently selected by both algorithms were defined as hub genes.

### 2.6. Validation of Hub Genes

Receiver operating characteristic (ROC) curves were plotted for each hub gene in the discovery cohort (26 samples) using the *pROC* R package to assess classification performance. Expression differences between DCM and control groups were evaluated by the Wilcoxon rank-sum test. External validation used two independent RNA-seq datasets: GSE161931 (5 DCM, 5 Con) and GSE274500 (4 DCM, 4 Con), both GPL24247, Illumina NovaSeq 6000, merged for joint analysis. Raw counts were filtered (CPM ≥ 1 in ≥3 samples), normalized using the voom method with quantile normalization (*edgeR/limma*), and batch effects were corrected with removeBatchEffect (*limma*) while preserving disease status information. ROC and Wilcoxon tests were repeated in the merged validation cohort (9 DCM, 9 Con).

### 2.7. Functional Characterization of Hub Genes

To characterize biological pathways associated with each hub gene, samples in the discovery cohort were stratified into high- and low-expression groups by median gene expression. Differential expression between the two groups was assessed using *limma*. The resulting logFC-ranked gene lists were subjected to GSEA using *clusterProfiler*. Significantly enriched pathways were visualized as enrichment score plots.

### 2.8. Immune and Stromal Cell Infiltration Analysis

The immune and stromal microenvironment was characterized using the mMCPcounter algorithm, which estimates the relative abundance of both immune and stromal cell populations from murine transcriptomic data [27]. Differences in estimated cell-type scores between DCM and control groups were assessed by the Wilcoxon rank-sum test. Spearman correlation was performed between hub gene expression and cell-type abundance scores to evaluate associations with the microenvironment. Multiple testing correction was applied using the Benjamini–Hochberg method across all 15 cell types, separately for the group comparisons and for each hub gene.

### 2.9. Single-Cell RNA Sequencing Analysis

ScRNA-seq data from GSE290095 (1 DCM, 1 Con) were processed using *Seurat* (v5) [28]. For each sample independently, data were normalized, dimensionality reduction and clustering were performed, and doublets were identified and removed using *DoubletFinder* [29]. The two samples were then merged for integration across conditions, and cells were retained based on the following quality control criteria: 500 ≤ nFeature_RNA ≤ 7500, nCount_RNA > 500 and <50,000, percent.mt ≤ 25%, and percent. HB ≤ 1%. The merged data were integrated using Harmony to facilitate joint clustering. UMAP dimensionality reduction was applied, and clustering resolution was determined by clustree analysis. Cell types were annotated manually based on canonical marker genes, and hub gene expression was visualized using violin plots. In silico gene knockout simulation was performed in DCM cardiomyocytes using *scTenifoldKnk* [30]. The manifold distance between the wild-type and KO networks was computed for each gene, and the top 20 genes with the largest manifold distance were retained as the genes most strongly affected by the predicted network perturbation.

### 2.10. Spatial Transcriptomics Analysis

Spatial transcriptomic data from GSE290094 (10× Visium FFPE; 4 Con, 3 DCM) were processed using Seurat with SCTransform normalization. Hub gene spatial expression was visualized using *SpatialFeaturePlot*, and spot-level expression was compared between control and DCM groups using the Wilcoxon rank-sum test. A ferroptosis driver score was computed per spot using *AddModuleScore* based on the FerrDb V2 driver gene set. Spot-level Spearman correlation was performed between hub gene expression and ferroptosis driver scores. Analyses were stratified by disease group to evaluate spatial co-associations.

### 2.11. Experimental Validation In Vivo

#### 2.11.1. Animal Studies and Ethics

All animal experiments were approved by the Animal Ethics Committee of Wuxi People’s Hospital Affiliated to Nanjing Medical University (Approval No. DL2025016) and were conducted in accordance with institutional guidelines. Male Sprague-Dawley rats (6–8 weeks old) were acclimated for 1 week and then randomly assigned to a control group (*n* = 6) or a DCM group (*n* = 6). The control group received a standard diet (MD17122, Jiangsu Medison, Yangzhou, China), while the DCM group received a high-fat diet (D12492, Speford, Beijing, China). After 4 weeks on the high-fat diet, rats in the DCM group were fasted for 10 h and then given a single intraperitoneal injection of streptozotocin (STZ, 35 mg/kg; Sigma-Aldrich, St. Louis, MO, USA) dissolved in citrate buffer [31]; control rats received an equal volume of the vehicle. Rats with random blood glucose ≥ 16.7 mmol/L at 72 h after injection were classified as diabetic. All animals were maintained for an additional 12 weeks before sacrifice [32]. Hearts were excised; portions were fixed in 4% paraformaldehyde, and the remainder were snap-frozen and stored at −80 °C.

Body weight and random blood glucose were monitored every two weeks throughout the observation period. At study endpoint, an oral glucose tolerance test (OGTT) was performed after an 8-h fast; glucose (2 g/kg) was administered by oral gavage and tail-vein blood glucose was measured at 0, 30, 60, 90 and 120 min.

#### 2.11.2. Transthoracic Echocardiography

Under isoflurane anesthesia, transthoracic echocardiography was performed with a high-resolution small-animal ultrasound system (Vevo 2100, VisualSonics, Toronto, ON, Canada). Left ventricular ejection fraction (LVEF), left ventricular fractional shortening (LVFS), left ventricular end-diastolic diameter (LVIDd), and left ventricular end-systolic diameter (LVIDs) were measured from M-mode images at the papillary muscle level.

#### 2.11.3. Histological Analyses

Fixed heart tissues were embedded in paraffin and sectioned at 5-µm thickness. Hematoxylin and eosin (H&E) staining was used to assess myocardial morphology, Masson’s trichrome staining to evaluate collagen deposition, and wheat germ agglutinin (WGA) staining to delineate cardiomyocyte cross-sectional area. Fibrotic area and cardiomyocyte cross-sectional area were quantified using ImageJ software (version 1.54g, National Institutes of Health, Bethesda, MD, USA).

#### 2.11.4. Transmission Electron Microscopy

After collection, cardiac tissue samples were fixed in 1% glutaraldehyde for 10 min, then in 3% glutaraldehyde at room temperature for 1 h. Samples were post-fixed in 1% osmium tetroxide in 0.1 mol/L cacodylate buffer at room temperature for 1 h and embedded in resin. Ultrathin sections were stained with uranyl acetate and lead nitrate and examined by transmission electron microscopy (TEM) to assess mitochondrial morphology and striated muscle organization.

#### 2.11.5. Quantitative Real-Time PCR

Total RNA was extracted from heart tissues with Trizol (Invitrogen, Carlsbad, CA, USA). Complementary DNA was synthesized using HiScript II Q RT SuperMix (Vazyme, Nanjing, China). Quantitative PCR was carried out with ChamQ Universal SYBR qPCR Master Mix (Vazyme, Nanjing, China) on a quantitative PCR instrument (Bio-Rad Laboratories, Hercules, CA, USA). Relative expression was calculated by the 2^−ΔΔCt^ method with β-actin as internal reference.

#### 2.11.6. Western Immunoblotting

Proteins were extracted from tissue samples using a protein extraction reagent, and concentrations were measured with a BCA protein assay kit (23,227, Thermo Scientific, Waltham, MA, USA). Proteins were resolved by SDS-PAGE and transferred to PVDF membranes. Membranes were blocked in 5% nonfat milk at room temperature for 2 h and incubated with the appropriate primary antibody overnight at 4 °C. After three washes with TBST, membranes were incubated with the secondary antibody for 2 h. Primary antibodies included anti-ACOT1 (acyl-CoA thioesterase 1) (1:1000, GB112157, Servicebio, Wuhan, China), anti-TXNIP (thioredoxin-interacting protein) (1:2000, 18243-1-AP, Proteintech, Wuhan, China), anti-GPX4 (1:1000, GB154327-100, Servicebio), anti-ACSL4 (1:1000, 22401-1-AP, Proteintech), anti-FTH1 (1:1000, 60875-5-Ig, Proteintech), and β-actin (HRP-60008; 1:1000; Proteintech). Bands were visualized with a chemiluminescent reagent (WBKLS0500, Millipore, Burlington, MA, USA) and quantified with ImageJ software. Aliquots of the same cardiac lysates were resolved on parallel gels, and each membrane was probed for a single target protein together with β-actin as its own loading control.

### 2.12. Statistical Analysis

Statistical analyses were performed separately for the bioinformatic and experimental components of this study. For the bioinformatic analyses, all computations were carried out in R (version 4.3.3). Two-group comparisons of transcriptomic measures were performed using the Wilcoxon rank-sum test unless otherwise specified, and multiple testing correction was applied using the Benjamini–Hochberg (BH) method where applicable. Figures were generated with *ggplot2* and assembled in Adobe Illustrator (version 2019, Adobe Inc., San Jose, CA, USA). For the experimental data, analyses were conducted in SPSS (version 29.0.2, IBM Corporation, Armonk, NY, USA), and results are presented as mean ± SEM. Normality and homogeneity of variance were assessed before comparison; two-group comparisons used the independent-samples *t*-test when these assumptions were met, and the Mann–Whitney U test otherwise. A two-tailed *p* < 0.05 was considered statistically significant throughout.

## 3. Results

### 3.1. Myocardium in DCM Exhibits a Ferroptosis-Associated Metabolic Remodeling Signature

**Figure 2:**
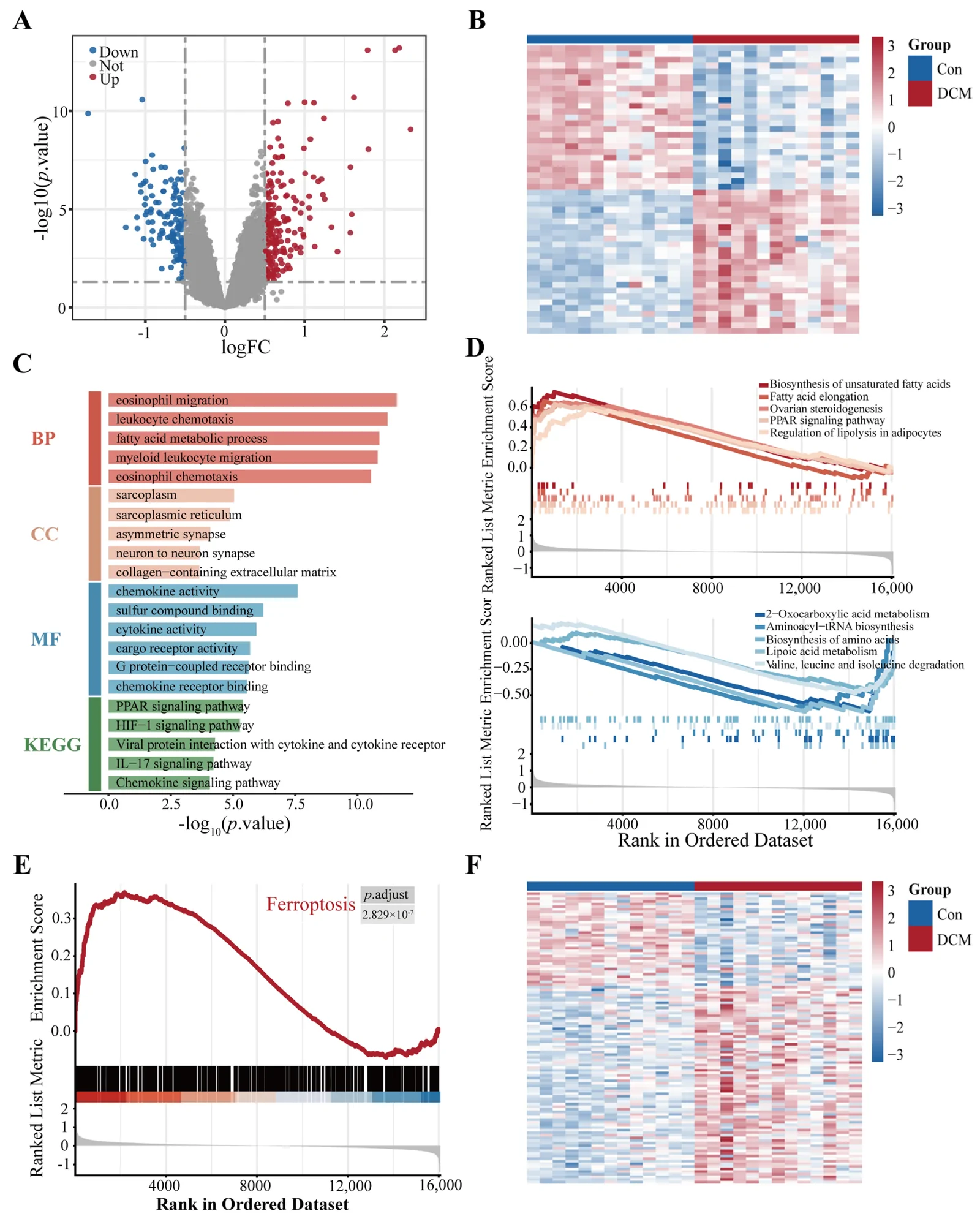
Identification of DEGs and ferroptosis-related alterations in diabetic cardiomyopathy. (**A**) Volcano plot of DEGs between Con and DCM samples. (**B**) Heatmap of DEGs across Con and DCM samples. (**C**) GO and KEGG enrichment analysis of DEGs. (**D**) GSEA of the top enriched KEGG pathways in DCM. (**E**) GSEA enrichment plot for the ferroptosis gene set. (**F**) Heatmap of ferroptosis-related gene expression in Con and DCM samples. Con—control; DCM—diabetic cardiomyopathy; DEG—differentially expressed gene; GSEA—gene set enrichment analysis.

To characterize the transcriptomic landscape of myocardium in DCM, three independent murine datasets (GSE123975, GSE155377, and GSE210611) were integrated into a single discovery cohort of 13 DCM and 13 Con samples. Inter-study batch effects were corrected using ComBat normalization prior to downstream analysis. Differential expression analysis yielded 317 DEGs (*p* < 0.05, |log_2_FC| > 0.5; Figure 2A). Given the modest sample size and the exploratory nature of this transcriptomic screen, this nominally significant threshold was applied to maximize candidate retention for downstream multistep filtering, rather than as a standalone criterion for gene selection. A BH-adjusted *p* < 0.05 threshold yielded 261 DEGs, and all six FRDEGs identified below remained significant under this criterion (Table S1). The top 25 upregulated and top 25 downregulated DEGs displayed distinct expression profiles that separated DCM from control myocardium (Figure 2B).

GO and KEGG enrichment analyses highlighted biological processes associated with immune cell chemotaxis, fatty acid metabolism, and chemokine/cytokine activity, alongside PPAR signaling, HIF-1 signaling, and the chemokine signaling cascade (Figure 2C). GSEA revealed a coordinated metabolic remodeling pattern in DCM myocardium: lipid anabolic pathways (biosynthesis of unsaturated fatty acids, fatty acid elongation, and PPAR signaling) were positively enriched in DCM, while amino acid catabolic processes (valine, leucine and isoleucine degradation, aminoacyl-tRNA biosynthesis) were negatively enriched (Figure 2D). Consistent with this pattern, GSEA of a FerrDb-derived ferroptosis gene set demonstrated significant positive enrichment in DCM samples (*p* = 2.829 × 10^−7^; Figure 2E), and expression profiling of ferroptosis-related genes across all samples revealed a distinct group-specific expression pattern consistent with altered ferroptosis-related transcriptional regulation in DCM myocardium (Figure 2F).

### 3.2. Identification of DCM-Associated Co-Expression Modules by WGCNA

**Figure 3:**
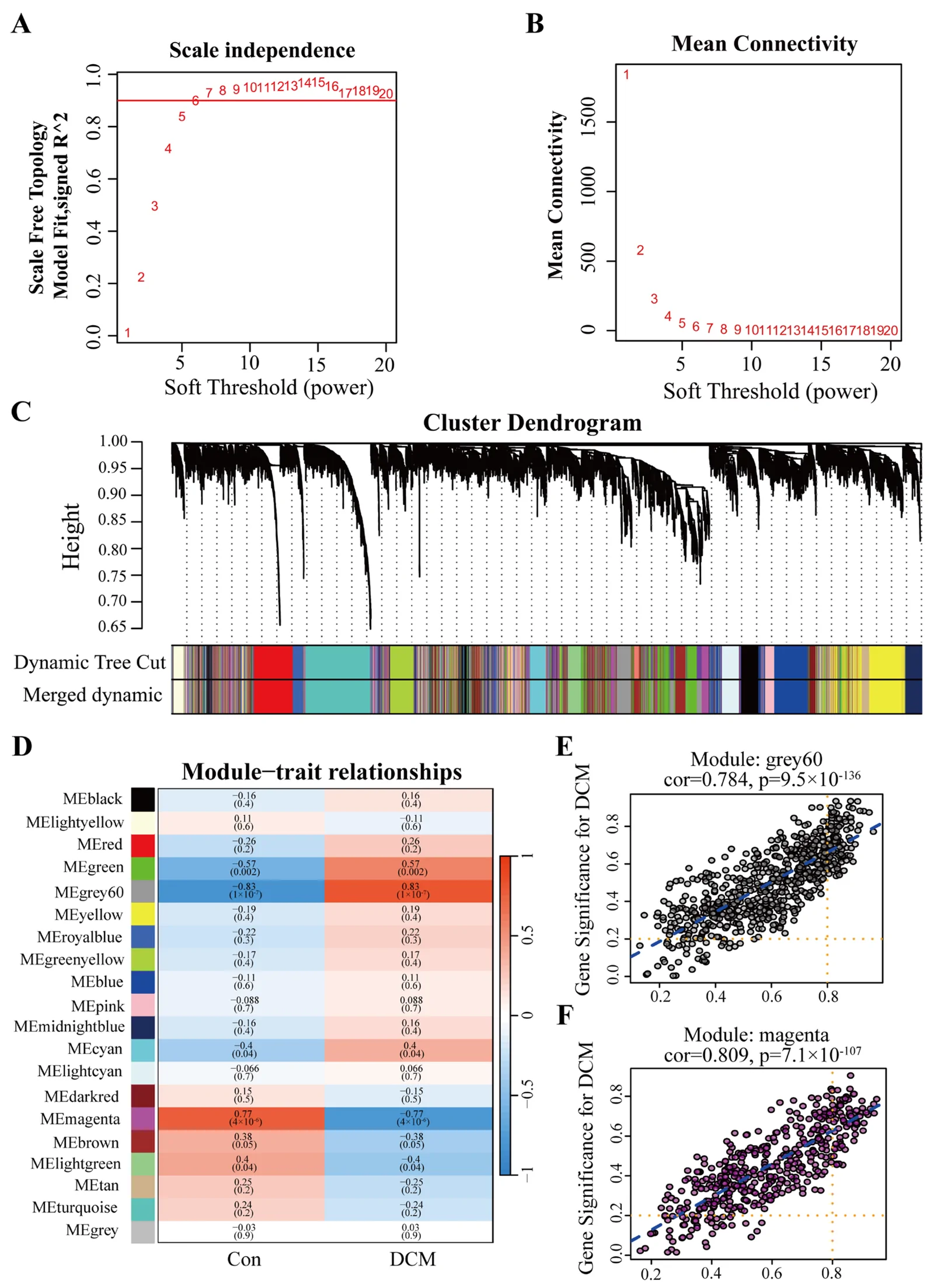
Weighted gene co-expression network analysis identified key modules associated with diabetic cardiomyopathy. (**A**,**B**) Selection of different soft-thresholding powers; the red horizontal line indicates the R^2^= 0.90 cutoff. (**C**) Gene clustering dendrogram with co-expression modules identified by dynamic tree cutting and merging; module colors in (**C**) correspond to module labels in (**D**). (**D**) Heatmap of module–trait correlations between module eigengenes and disease status (Con vs. DCM); values in (**D**) are correlation coefficients with *p* values in parentheses. (**E**,**F**) Scatter plots of gene significance (GS) versus module membership (MM) in the grey60 and magenta modules; dots in (**E**,**F**) represent individual genes. Con—control; DCM—diabetic cardiomyopathy; GS—gene significance; MM—module membership.

To identify disease-associated co-expression modules within the DCM transcriptome, WGCNA was applied to the batch-corrected expression matrix. A soft-thresholding power of β = 6 was selected to satisfy the scale-free topology criterion (R^2^ ≥ 0.90; Figure 3A,B). Hierarchical clustering with dynamic tree cutting resolved 19 non-grey co-expression modules from the input gene set (Figure 3C). Module–trait correlation analysis identified grey60 (646 genes; cor = 0.83, *p* = 1 × 10^−7^) and magenta (457 genes; cor = −0.77, *p* = 4 × 10^−6^) as the modules with the highest absolute correlation with DCM status (Figure 3D). Within both modules, gene significance showed strong positive associations with module membership, indicating that genes highly connected within each module were also strongly associated with DCM phenotype (Figure 3E,F). The combined 1103 genes from these two modules were carried forward as WGCNA-prioritized candidates for downstream intersection analysis.

### 3.3. Identification of Ferroptosis-Related Hub Genes

**Figure 4:**
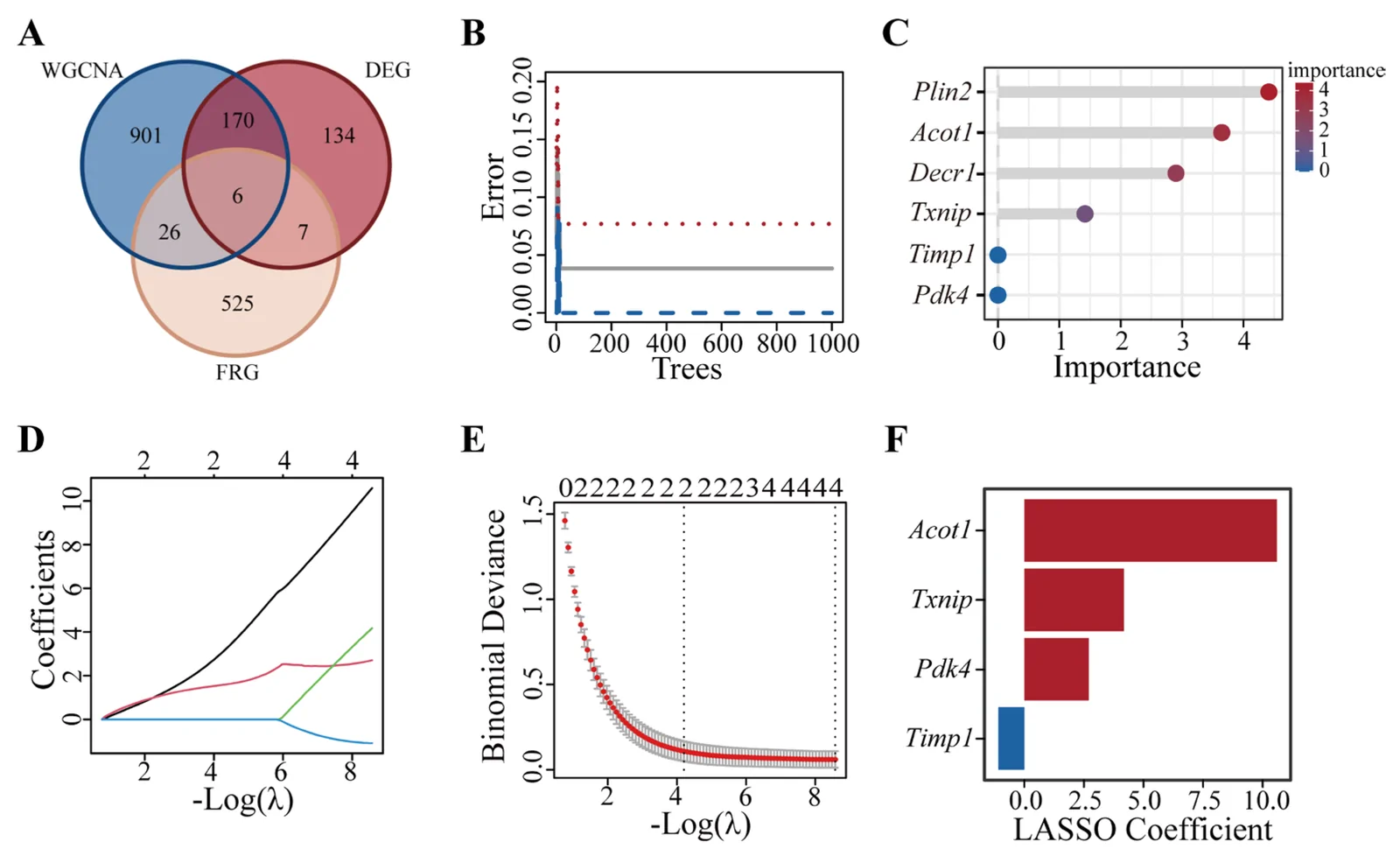
Screening of ferroptosis-related signature genes using machine learning algorithms. (**A**) Venn diagram showing the overlap among WGCNA hub module genes, DEGs, and ferroptosis-related genes (FRGs). (**B**) Random forest error plot showing model performance as the number of trees increases; dashed red, solid grey and dashed blue lines indicate DCM, out-of-bag and control error rates. (**C**) Variable importance plot of candidate genes generated by the random forest model; dot color indicates importance score. (**D**) LASSO coefficient profiles of candidate genes; each colored line represents one candidate gene. (**E**) 5-fold cross-validation for tuning parameter selection in the LASSO model. (**F**) Bar plot showing the coefficients of genes retained in the LASSO model. Con—control; DCM—diabetic cardiomyopathy; DEG—differentially expressed gene.

To prioritize ferroptosis-related hub genes from the candidate pool, the intersection of the 317 DEGs, the 1103 WGCNA-prioritized module genes, and the 564 FerrDb ferroptosis regulators yielded 6 genes meeting all three criteria, designated as ferroptosis-related differentially expressed genes (FRDEGs): *Plin2*, *Acot1*, *Decr1*, *Txnip*, *Pdk4*, and *Timp1* (Figure 4A).

These 6 FRDEGs were subsequently subjected to two independent machine learning approaches for further selection. Random Forest classification ranked the six candidates according to variable importance, with *Plin2*, *Acot1*, *Decr1*, and *Txnip* contributing most strongly to the classification model (Figure 4B,C). LASSO logistic regression with cross-validation identified four genes retaining non-zero coefficients at the optimal penalty parameter: *Acot1*, *Txnip*, *Pdk4*, and *Timp1* (Figure 4D–F). The overlap between LASSO- and RF-selected genes identified *Acot1* and *Txnip* as consensus hub gene candidates consistently prioritized by both algorithms, and these two genes were carried forward for all subsequent analyses.

Hypergeometric testing against the WGCNA input gene set (N = 9071) showed significant enrichment for the DEG–WGCNA overlap (observed = 176, expected = 38.6, 4.57-fold, *p* < 10^−50^) and the WGCNA–FerrDb overlap (observed = 32, expected = 22.6, 1.41-fold, *p* = 0.026). The three-way intersection was not significantly enriched (observed = 6, expected = 3.6, *p* = 0.153), indicating that this step functions as a biologically directed candidate filter rather than a statistically enriched selection. Global enrichment of ferroptosis-related transcriptional programmes in DCM myocardium is instead supported by the ferroptosis gene-set GSEA result (*p* = 2.83 × 10^−7^; Figure 2E).

### 3.4. Classification Performance and External Validation

**Figure 5:**
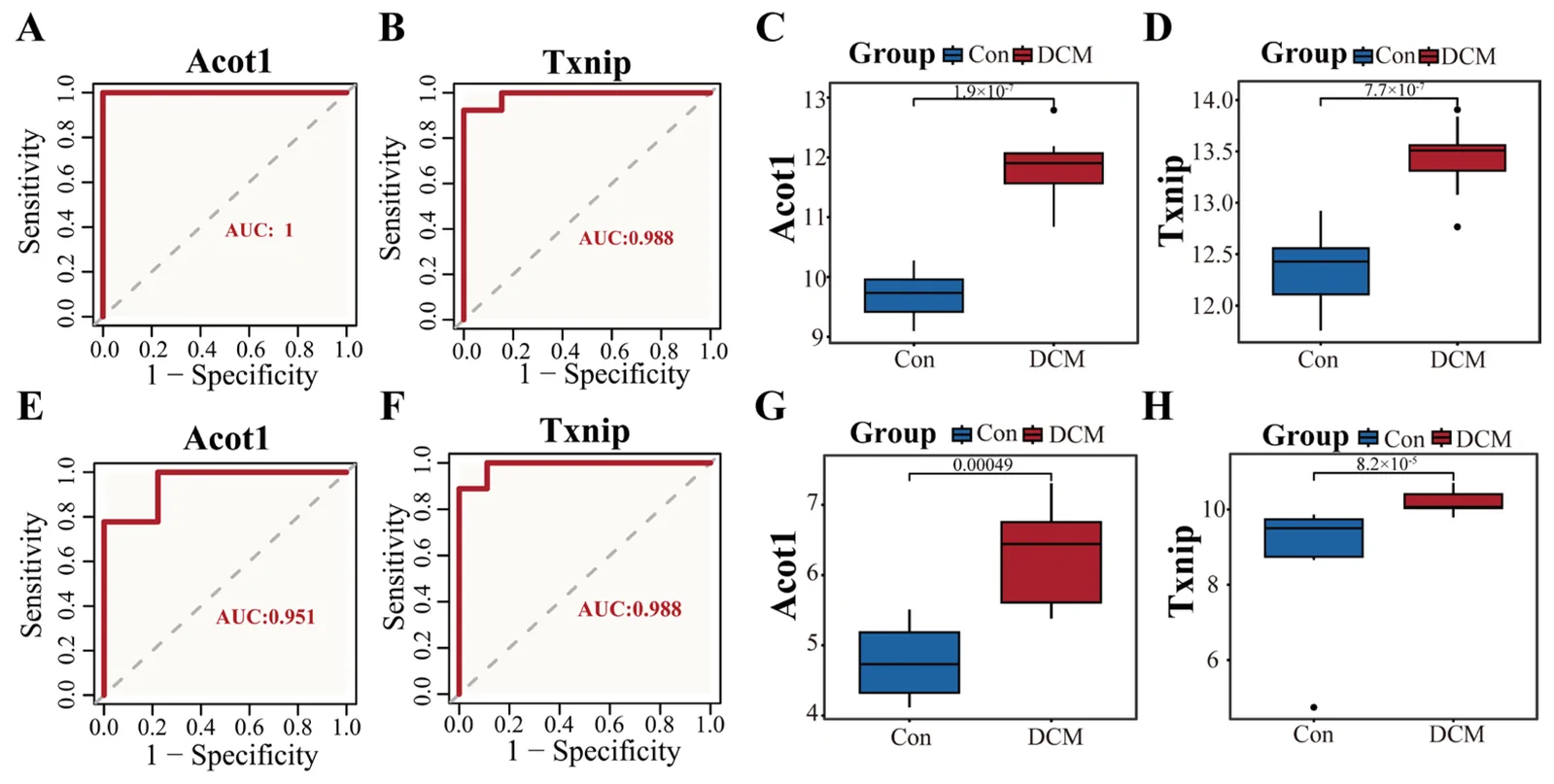
Expression patterns and classification performance of *Acot1* and *Txnip* in the training and validation cohorts. (**A**,**B**) ROC curves of *Acot1* (AUC = 1.000) and *Txnip* (AUC = 0.988) in the training cohort. (**C**,**D**) Expression levels of *Acot1* and *Txnip* in Con and DCM samples from the training cohort (Wilcoxon test). (**E**,**F**) ROC curves of *Acot1* (AUC = 0.951) and *Txnip* (AUC = 0.988) in the external validation cohort. (**G**,**H**) Expression levels of *Acot1* and *Txnip* in Con and DCM samples from the external validation cohort (Wilcoxon test). Con—control; AUC—area under the curve; DCM—diabetic cardiomyopathy; ROC—receiver operating characteristic.

The discriminatory performance of *Acot1* and *Txnip* was evaluated by ROC analysis in the discovery cohort. *Acot1* achieved an AUC of 1.000 and *Txnip* an AUC of 0.988 (Figure 5A,B). As both genes were selected using this same dataset, these values represent in-sample estimates and are presented as descriptive rather than unbiased performance metrics. Both genes were significantly upregulated in DCM myocardium (*Acot1*: *p* = 1.9 × 10^−7^; *Txnip*: *p* = 7.7 × 10^−7^; Figure 5C,D).

To assess generalizability, external validation was performed in an independent cohort of 9 DCM and 9 Con samples merged from GSE161931 and GSE274500. Both hub genes maintained high ROC performance in this cohort (*Acot1*: AUC = 0.951; *Txnip*: AUC = 0.988; Figure 5E,F), and their upregulation in DCM was reproduced at the expression level (*Acot1*: *p* = 4.9 × 10^−4^; *Txnip*: *p* = 8.2 × 10^−5^; Figure 5G,H). These external validation AUC estimates, derived from an independent cohort profiled by a different sequencing platform (RNA-seq vs. microarray), constitute the primary evidence for the cross-dataset reproducibility of the identified signatures.

### 3.5. Functional Characteristics and Immune Associations of Hub Genes

Given that hub genes were selected for disease association, expression-based stratification by hub gene median largely recapitulates the DCM/Con dichotomy (*Acot1*: complete concordance, κ = 1.0; *Txnip*: 92.3% concordance, κ = 0.846). The following GSEA results therefore serve as descriptive functional contextualization of the hub gene expression pattern rather than as independent evidence of biological function.

**Figure 6:**
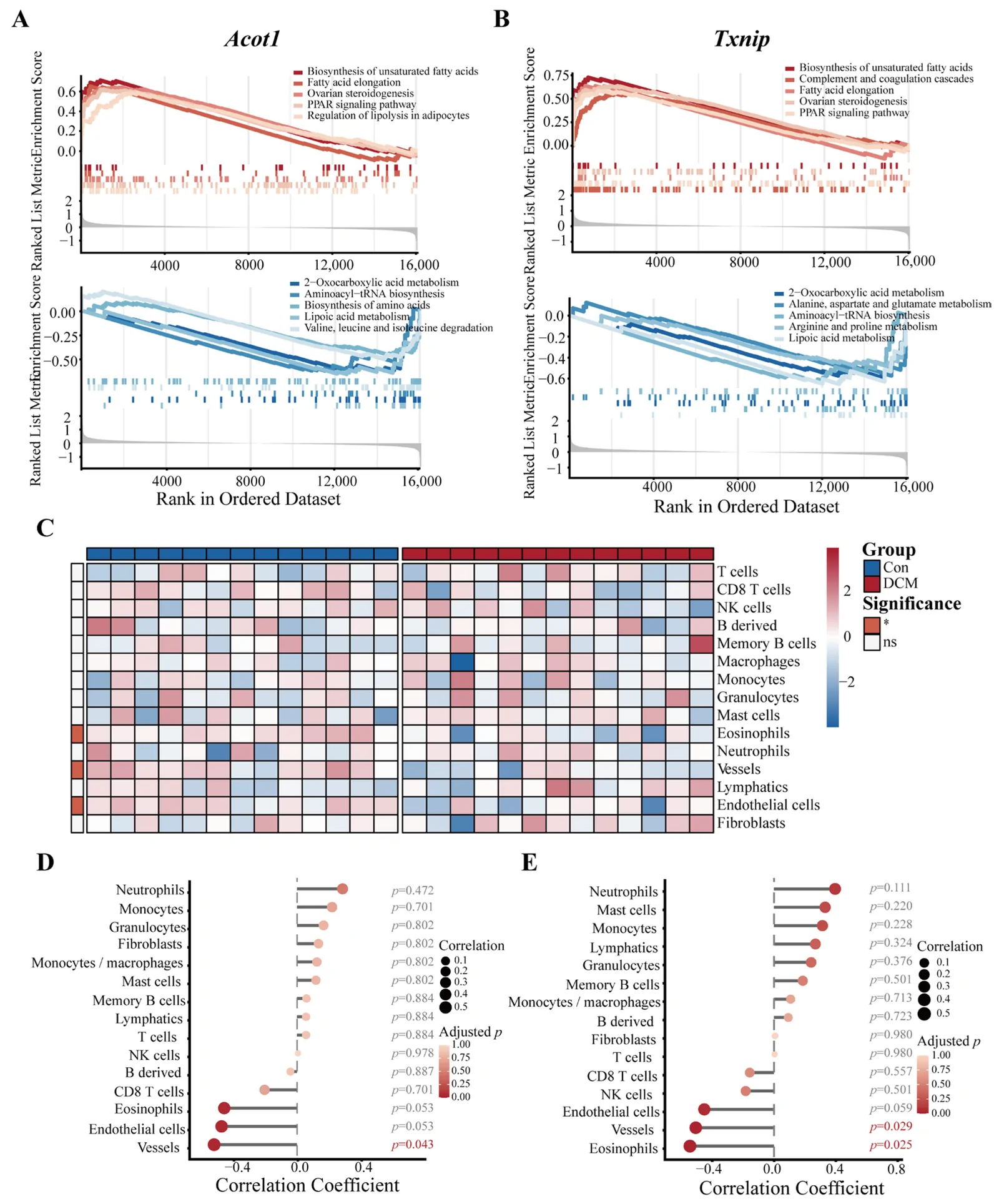
Functional characteristics and immune associations of *Acot1* and *Txnip* in diabetic cardiomyopathy. (**A**,**B**) Single-gene GSEA of samples stratified by *Acot1* (**A**) and *Txnip* (**B**) expression, showing top enriched and depleted KEGG pathways. (**C**) Heatmap of immune cell infiltration estimated by mMCPcounter across Con and DCM samples. (**D**,**E**) Correlation between *Acot1* (**D**) and *Txnip* (**E**) expression and infiltrating immune cell populations; significant correlations (adj. *p* < 0.05) are highlighted in red. Con—control; DCM—diabetic cardiomyopathy; GSEA—gene set enrichment analysis; * —adjusted *p* < 0.05; ns, not significant.

To characterize the functional context of each hub gene, single-gene GSEA was performed by stratifying samples into high- and low-expression groups based on median expression of each hub gene. For *Acot1*, lipid anabolic pathways (biosynthesis of unsaturated fatty acids, fatty acid elongation, PPAR signaling) were positively enriched in the high-expression group, while amino acid catabolic pathways (valine, leucine and isoleucine degradation, aminoacyl-tRNA biosynthesis) were negatively enriched (Figure 6A). *Txnip* showed a similar lipid–amino acid metabolic divergence, with complement and coagulation cascades additionally among the positively enriched pathways (Figure 6B). These results indicate that both hub genes are functionally associated with lipid metabolic networks in DCM.

Immune and stromal cell composition was estimated using the mMCPcounter algorithm, which quantifies the relative abundance of both immune and stromal cell populations. Comparison of estimated abundances between DCM and control groups revealed differences in eosinophils, endothelial cells, and vessels (all BH-adjusted *p* < 0.05; Figure 6C). Spearman correlation analysis with BH correction showed that *Acot1* expression was significantly correlated with vessels (rho = −0.561, adj. *p* = 0.043), and *Txnip* with eosinophils (rho = −0.586, adj. *p* = 0.025) and vessels (rho = −0.546, adj. *p* = 0.029) (Figure 6D,E). These findings suggest that hub gene expression is associated with the immune and stromal microenvironment in DCM myocardium, though the direction of causality remains to be established.

### 3.6. Single-Cell and Spatial Transcriptomic Characterization of Hub Genes

**Figure 7:**
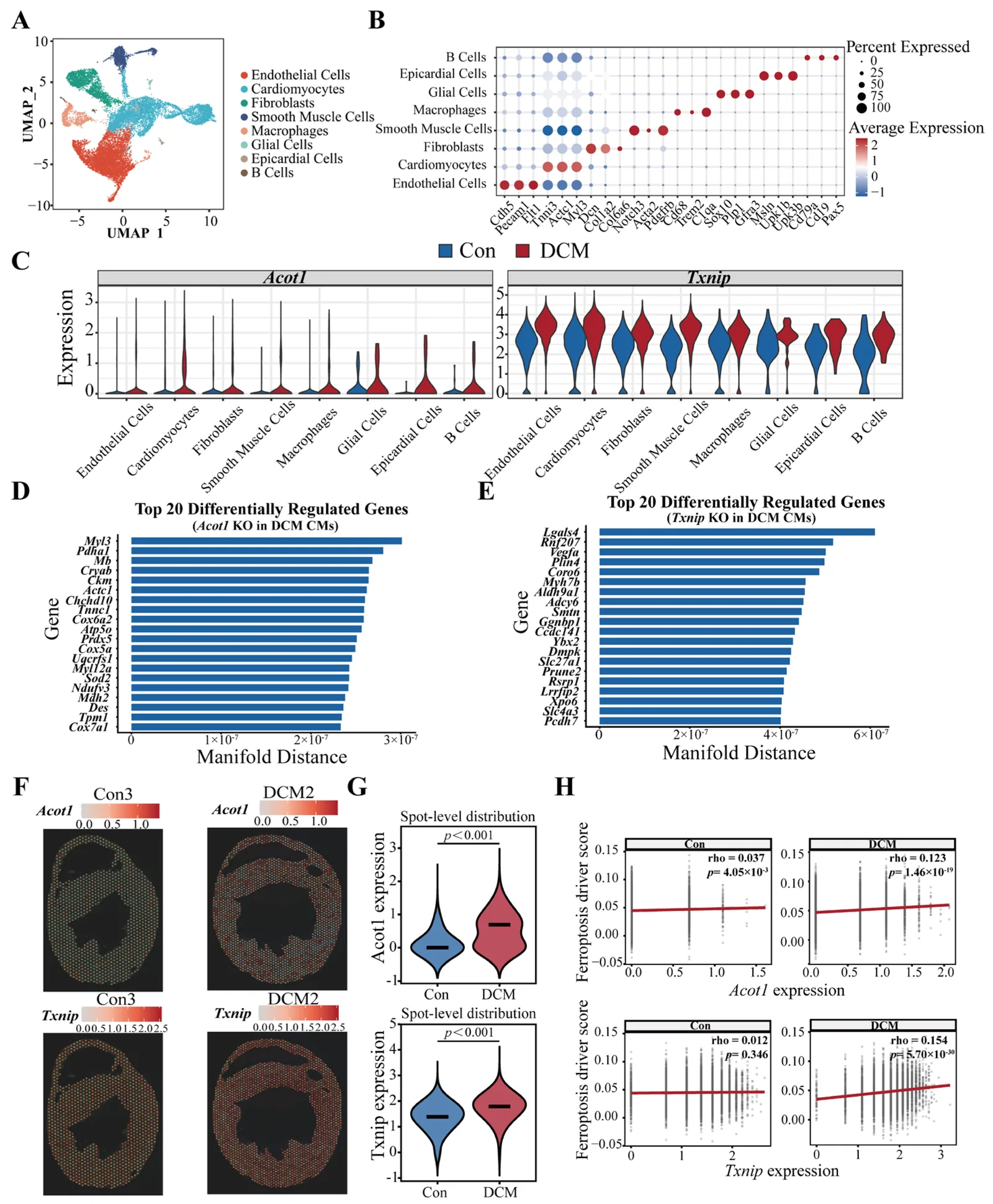
Single-cell and spatial transcriptomic characterization of *Acot1* and *Txnip* in diabetic cardiomyopathy. (**A**) UMAP plot showing major cell populations identified in the scRNA-seq dataset. (**B**) Dot plot of canonical marker genes used for cell-type annotation. (**C**) Violin plots showing expression distributions of *Acot1* and *Txnip* across cell types in Con and DCM samples. (**D**,**E**) Top 20 genes with the largest manifold distances after in silico knockout of *Acot1* (**D**) or *Txnip* (**E**) in DCM cardiomyocytes. (**F**) Spatial expression maps of *Acot1* and *Txnip* in representative Con and DCM myocardial sections. (**G**) Spot-level violin plots of *Acot1* and *Txnip* expression in Con and DCM samples. (**H**) Spearman correlation between AddModuleScore-derived ferroptosis driver scores and *Acot1* or *Txnip* expression in Con and DCM samples; red lines indicate linear regression fits. Con—control; DCM—diabetic cardiomyopathy; scRNA-seq—single-cell RNA sequencing; UMAP—uniform manifold approximation and projection.

To explore the cellular distribution of hub genes at single-cell resolution, scRNA-seq data from GSE290095 (1 DCM, 1 Con) were analyzed. Given the single biological replicate per condition, these findings provide exploratory evidence of cell-type-specific expression patterns. Unsupervised clustering identified eight major cardiac cell populations: endothelial cells, cardiomyocytes, fibroblasts, smooth muscle cells, macrophages, glial cells, epicardial cells, and B cells (Figure 7A,B). Violin plots showed that *Txnip* was broadly expressed across multiple cell types and appeared higher in DCM samples, whereas *Acot1* expression was more restricted, with notable levels in cardiomyocytes and endothelial cells, and a similar upward trend in DCM (Figure 7C).

To explore predicted network perturbations associated with hub gene loss in cardiomyocytes, in silico knockout of *Acot1* and *Txnip* was performed in the DCM cardiomyocyte subset using *scTenifoldKnk*. The top 20 genes most affected by *Acot1* knockout (ranked by manifold distance) included genes related to cardiac contractile and mitochondrial energetic machinery, such as sarcomeric components (*Myl3*, *Actc1*, *Tnnc1*, *Tpm1*, *Des*, *Myl12a*), oxidative phosphorylation subunits (*Cox5a*, *Cox6a2*, *Cox7a1*, *Atp5o*, *Uqcrfs1*, *Ndufv3*), and antioxidant/stress-response genes (*Cryab*, *Prdx5*, *Sod2*) (Figure 7D). In contrast, the top genes affected by *Txnip* knockout included those involved in lipid handling, vascular signaling, and ion transport, including *Plin4*, *Slc27a1*, *Vegfa*, *Adcy6*, *Slc4a3*, and *Myh7b* (Figure 7E).

Spatial transcriptomic analysis (GSE290094; 4 Con, 3 DCM) revealed visually elevated spatial expression of both hub genes in DCM myocardium relative to the control group (Figure 7F). Spot-level quantification confirmed significant upregulation of both hub genes in DCM compared with control tissue (*Acot1*: *p* < 0.001; *Txnip*: *p* < 0.001; Figure 7G). Spot-level Spearman correlation analysis revealed significant associations between hub gene expression and ferroptosis driver scores in DCM tissue (*Acot1*: rho = 0.123, *p* = 1.46 × 10^−19^; *Txnip*: rho = 0.154, *p* = 5.70 × 10^−30^), whereas correlations in control tissue were negligible (rho < 0.05; Figure 7H). Although the absolute correlation values are modest, consistent with signal dilution across heterogeneous Visium spots, the disease-specific directionality supports a spatial association between hub gene expression and ferroptosis activity in DCM myocardium.

### 3.7. Experimental Validation of ACOT1 and TXNIP In Vivo

**Figure 8:**
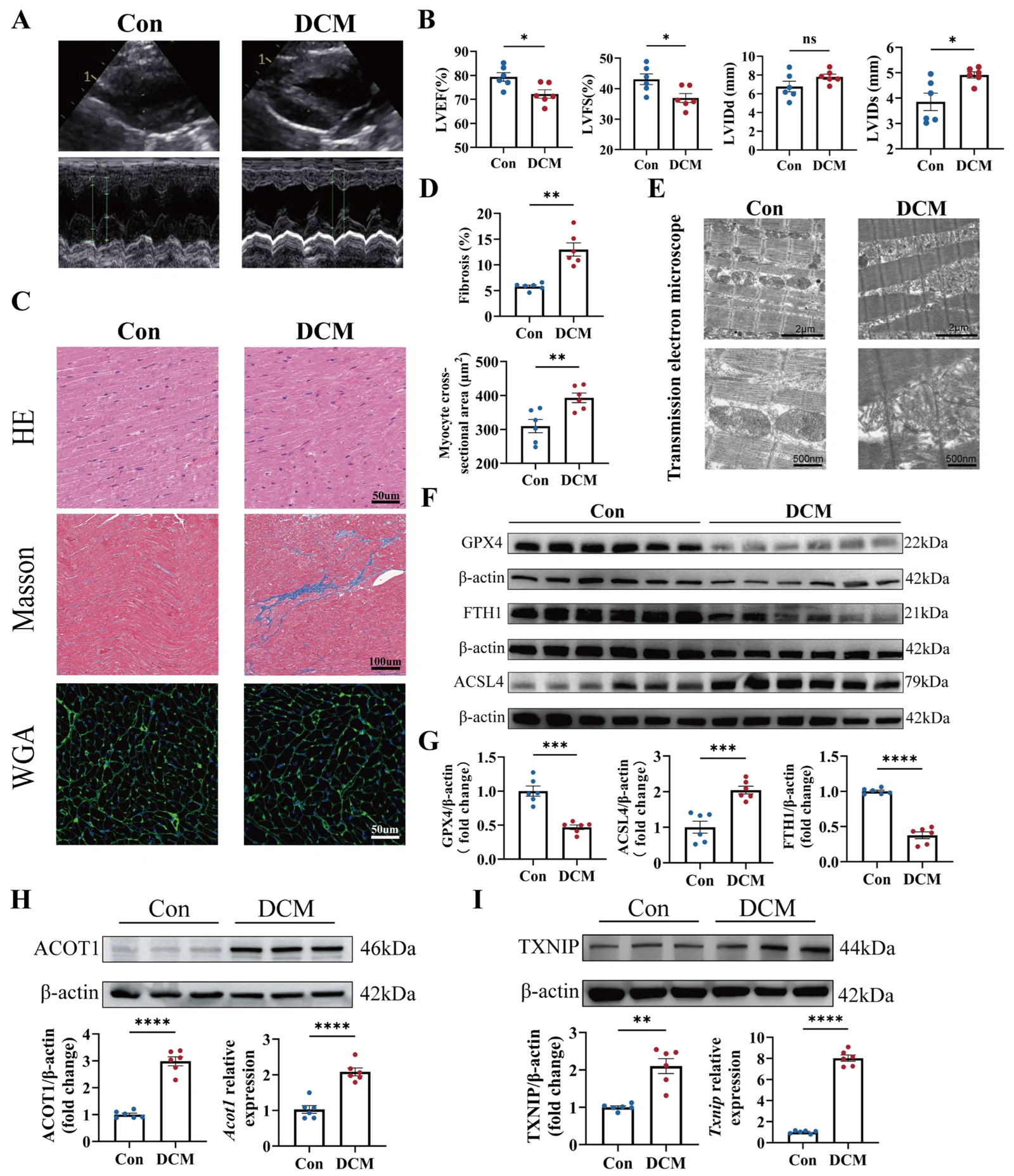
Experimental validation of ACOT1 and TXNIP upregulation in DCM rat model. (**A**) Representative two-dimensional and M-mode echocardiographic images of the left ventricle in Con and DCM rats. (**B**) Quantification of left ventricular ejection fraction (LVEF), fractional shortening (LVFS), end-diastolic internal diameter (LVIDd), and end-systolic internal diameter (LVIDs). (**C**) Representative hematoxylin–eosin (HE), Masson’s trichrome, and wheat germ agglutinin (WGA) staining of myocardial sections. (**D**) Quantification of interstitial fibrosis (%) and cardiomyocyte cross-sectional area (µm^2^). (**E**) Representative transmission electron microscopy images of myocardial ultrastructure in Con and DCM rats. (**F**) Representative Western blots of GPX4, ACSL4, and FTH1 in Con and DCM hearts. (**G**) Quantification of GPX4, ACSL4, and FTH1 protein levels. (**H**) Representative Western blot of ACOT1 and quantification of ACOT1 protein levels together with *Acot1* mRNA expression. (**I**) Representative Western blot of TXNIP and quantification of TXNIP protein levels together with *Txnip* mRNA expression. Con—control; DCM—diabetic cardiomyopathy; ns—not significant; * *p* < 0.05, ** *p* < 0.01, *** *p* < 0.001, **** *p* < 0.0001; Data are presented as mean ± SEM, with each dot representing an individual animal (blue, Con; red, DCM); *n* = 6 per group for all quantifications.

To validate the bioinformatic findings in vivo, a high-fat diet/streptozotocin (HFD/STZ)-induced DCM rat model was established. Body weight rose more steeply in the DCM group during high-fat feeding and plateaued thereafter, converging with control values by the end of the observation period (Figure S1A). Rats in the DCM group developed sustained hyperglycemia from week 6 onward (Figure S1B), and a terminal OGTT confirmed marked glucose intolerance, with blood glucose remaining above 20 mmol/L throughout the 120-min test compared with 5–9 mmol/L in control rats (Figure S1C). Echocardiographic assessment showed that DCM rats exhibited significantly reduced LVEF and LVFS, along with increased LVIDs compared with the control group (*p* < 0.05), consistent with impaired cardiac systolic function in the diabetic model (Figure 8A,B). H&E staining revealed well-organized myocardial fibers with intact transverse striations in control hearts, whereas the DCM group showed disordered myocardial architecture, myofiber swelling, nuclear pyknosis, and focal inflammatory cell infiltration (Figure 8C). Masson’s trichrome staining showed markedly increased collagen deposition and interstitial fibrosis in DCM hearts relative to the control group (*p* < 0.05; Figure 8C,D). WGA staining further demonstrated significantly enlarged cardiomyocyte cross-sectional area in DCM hearts compared with the control group (*p* < 0.05; Figure 8C,D), indicating cardiomyocyte hypertrophy. Transmission electron microscopy (TEM) further revealed ultrastructural alterations consistent with, but not specific to, ferroptosis, including shrunken mitochondria with increased membrane density, reduced cristae, and outer membrane rupture, whereas mitochondria in control hearts displayed intact cristae and normal morphology (Figure 8E).

Ferroptosis effector proteins were further assessed in the same cardiac lysates. Compared with control hearts, DCM hearts showed reduced GPX4 (*p* < 0.001), increased ACSL4 (*p* < 0.001), and reduced FTH1 (*p* < 0.0001) (Figure 8F,G). GPX4 depletion, ACSL4 enrichment and ferritin loss together constitute a ferroptosis-associated protein signature that provides biochemical context for the ultrastructural alterations observed by TEM.

RT-qPCR analysis showed that mRNA expression of both *Acot1* and *Txnip* were significantly upregulated in DCM rat cardiac tissue compared with the control group (*p* < 0.05; Figure 8H,I). Western blotting showed correspondingly elevated protein levels of both genes in DCM hearts (*p* < 0.05; Figure 8H,I). Taken together, these experimental results are consistent with the bioinformatic predictions, supporting the upregulation of ACOT1 and TXNIP as a reproducible feature of the DCM phenotype.

## 4. Discussion

DCM remains a significant clinical challenge due to its insidious progression and limited understanding of the molecular events driving cardiomyocyte injury. In this study, we employed an integrative bioinformatics strategy combining differential expression analysis, WGCNA, and dual machine learning algorithms to identify ferroptosis-related signature genes in DCM. Integrative filtering of disease-associated genes and ferroptosis-related candidates, followed by LASSO and RF convergence, prioritized *Acot1* and *Txnip* as hub genes. Both genes showed strong classification performance in the discovery cohort (AUC = 1.000 and 0.988), though these near-perfect values should be interpreted cautiously given the limited sample size (*n* = 26). Importantly, both genes maintained high discriminatory capacity in a fully independent external validation cohort using a different sequencing platform (RNA-seq vs. microarray; AUC = 0.951 and 0.988), supporting the robustness of the identified signatures. These findings were further contextualized by single-gene GSEA, immune infiltration profiling, single-cell and spatial transcriptomics, providing a multi-layered characterization of the ferroptosis–metabolism–microenvironment axis in DCM.

Functional enrichment analyses revealed a pronounced metabolic shift in DCM myocardium, characterized by upregulation of lipid metabolism-related pathways and concomitant suppression of amino acid metabolic pathways. The healthy heart derives approximately 70% of its ATP from fatty acid oxidation [7], and in diabetes, insulin resistance further increases fatty acid uptake while suppressing glucose utilization, resulting in lipotoxicity and heightened susceptibility to oxidative damage [6,7]. The reciprocal downregulation of amino acid catabolism is consistent with this substrate switch toward lipid reliance. Enrichment of the HIF-1 signaling pathway further suggests that hypoxia-driven transcriptional responses may contribute to ferroptosis susceptibility in this setting. Within this remodeled metabolic landscape, the two hub genes occupy complementary nodes: *Acot1* governs fatty acyl-CoA handling in the lipid-overloaded myocardium, whereas *Txnip* couples glucose sensing to thioredoxin-dependent redox control. Together, these functions position lipid metabolism and oxidative stress as converging upstream inputs to ferroptosis, consistent with the central roles of *Acot1* and *Txnip* in DCM.

### 4.1. ACOT1: Upregulation in DCM and Its Potential Link to Ferroptosis Resistance

ACOT1 is a cytosolic enzyme that catalyzes the hydrolysis of long-chain fatty acyl-CoAs to free fatty acids and coenzyme A, thereby modulating the balance between esterified and free fatty acid pools [33]. In our study, ACOT1 was significantly upregulated in DCM myocardium compared with the control group, a finding consistently observed across the discovery cohort, the external validation cohort, and the spatial transcriptomic dataset. This upregulation pattern is consistent with the observation by Yang et al. [34], who reported an approximately 11- to 12-fold increase in ACOT1 expression in the myocardium of db/db diabetic mice. In that study, *Acot1* overexpression attenuated diabetic cardiac dysfunction by repressing the PPARα/PGC1α signaling axis, reducing ROS generation and MDA accumulation, and reversing the maladaptive shift toward exclusive fatty acid oxidation [34]. These findings raise the possibility that ACOT1 upregulation in DCM may represent an adaptive response to the metabolic and oxidative stress imposed by the diabetic milieu, although this interpretation remains to be directly tested through gain- and loss-of-function approaches in the DCM context.

Evidence from other cardiac injury models further supports a potential anti-ferroptotic role for ACOT1. Liu et al. [35] demonstrated that *Acot1* overexpression in cardiomyocytes protects against doxorubicin-induced ferroptosis by reshaping cellular lipid composition and reducing the pool of polyunsaturated fatty acyl-CoAs available for peroxidation. Conversely, *Acot1* knockdown sensitized cardiomyocytes to ferroptotic death. Mechanistically, ACOT1 catalyzes the reaction opposite to that of ACSL4, a well-established pro-ferroptotic enzyme, suggesting that the balance between ACOT1 and ACSL4 activities may influence ferroptosis susceptibility in cardiomyocytes. Beyond its lipid-modulating function, ACOT1 has been implicated in broader cardioprotective pathways: Heden et al. [36] showed that global *Acot1*-knockout mice exhibit increased energy expenditure via UCP2-mediated mitochondrial uncoupling, and Hou et al. [37] demonstrated that EGR2-mediated transcriptional suppression of *Acot1* exacerbates cardiac inflammation and apoptosis in pressure-overload heart failure. Collectively, these independent lines of evidence suggest that ACOT1 operates at the intersection of lipid metabolism, ferroptosis resistance, and mitochondrial homeostasis in the heart. Our data, showing consistent upregulation of ACOT1 across multiple independent DCM datasets and experimental models, are compatible with a protective role. However, an alternative interpretation, that sustained *Acot1* upregulation contributes to excessive free fatty acid release and lipotoxicity, cannot be excluded. Definitive resolution of this question will require targeted functional experiments in DCM models. Consistent with this functional context, in silico *Acot1* knockout in DCM cardiomyocytes preferentially perturbed sarcomeric (e.g., *Myl3*, *Actc1*, *Tnnc1*, *Des*) and oxidative phosphorylation (e.g., *Cox5a*, *Atp5o*, *Ndufv3*) gene programs (Figure 7D), consistent with a possible association between ACOT1-related network perturbation and cardiomyocyte contractile and energetic programs under metabolic stress.

### 4.2. TXNIP: A Hyperglycemia-Responsive Protein with Emerging Links to Ferroptosis

TXNIP directly binds to and inhibits the major cellular antioxidant thioredoxin (TRX), thereby amplifying intracellular oxidative stress. Beyond this canonical role, TXNIP functions as an upstream activator of the NLRP3 inflammasome, promotes ER stress-mediated apoptosis, and participates in multiple cell death pathways [38]. In the context of diabetes, *Txnip* is among the most robustly upregulated genes in tissues exposed to hyperglycemia and is recognized as a mediator of glucose-induced cellular injury [38]. Our finding that TXNIP is significantly upregulated in DCM myocardium is therefore consistent with the hyperglycemia-driven pathological cascade characteristic of diabetic complications. This interpretation is further supported by a recent study showing that PBX1 overexpression attenuated oxidative stress and apoptosis in DCM through transcriptional inhibition of *Txnip* [39].

Emerging evidence has linked TXNIP to ferroptosis regulation. Nagakannan et al. [40] demonstrated that TXNIP promotes ferroptosis through NCOA4-mediated ferritinophagy, elevating cytosolic labile iron levels and amplifying lipid peroxidation. Notably, this pro-ferroptotic function was shown to be independent of the GSH/GPX4 axis, suggesting that TXNIP may operate through a parallel, iron-centric ferroptosis pathway [40]. In cardiomyopathy-related contexts, the ATF4/DDIT4/TXNIP axis has been identified as a regulatory circuit of interest. Chen et al. [41] demonstrated that in septic cardiomyopathy, ATF4 transcriptionally activates *Ddit4*, which promotes TXNIP-mediated ferroptosis and mitochondrial dysfunction. Pharmacological inhibition of ATF4 with ISRIB suppressed this cascade and improved cardiac function. Although this axis was characterized in septic rather than diabetic cardiomyopathy, the shared downstream effectors, including mitochondrial dysfunction, oxidative stress, and iron-dependent cell death, are also central to DCM pathogenesis, raising the possibility that similar regulatory mechanisms may operate in the diabetic heart.

Taken together, these findings from independent studies support TXNIP as a biologically plausible contributor linking hyperglycemia, oxidative stress, and ferroptosis-prone states in cardiomyocytes. However, direct evidence for a causal role of TXNIP in ferroptosis within the DCM context is currently lacking, and targeted loss-of-function experiments will be required to determine whether *Txnip* inhibition can attenuate ferroptotic cardiomyocyte death in the diabetic myocardium.

Notably, the concurrent reduction of FTH1 protein and upregulation of TXNIP observed in DCM myocardium is consistent with the NCOA4-mediated ferritinophagy mechanism described by Nagakannan et al. [40], providing directional support for the relevance of this pathway in the diabetic heart. ACOT1 and TXNIP further occupy catalytically opposing positions with respect to acyl-CoA metabolism: TXNIP promotes ferroptotic susceptibility, whereas ACOT1 may limit the polyunsaturated fatty acyl-CoA pool that supports ACSL4-dependent lipid peroxidation. The concurrent increase in ACSL4 protein suggests that ACOT1 upregulation, although present, did not offset the pro-ferroptotic shift. One plausible interpretation is that the co-upregulation of both genes reflects a pro-ferroptotic milieu accompanied by a partial compensatory response.

### 4.3. Microenvironment Alterations Associated with Hub Gene Expression

Immune infiltration analysis using mMCPcounter revealed significant differences in estimated eosinophil, vessel, and endothelial cell scores between DCM and control groups. Spearman correlation analysis further showed that both hub genes were inversely associated with vessel scores, with *Txnip* additionally associated with eosinophils. As DCM samples exhibit higher hub gene expression alongside lower estimated cell scores, these pooled correlations largely reflect disease-group differences rather than within-group gene–cell relationships. Although the mMCPcounter algorithm estimates both immune and stromal components from bulk expression data, and the observed correlations do not establish causality, these associations are consistent with the growing recognition that ferroptotic cell death is inherently immunogenic. Dying cells undergoing ferroptosis release damage-associated molecular patterns (DAMPs) and oxidized phospholipids that activate innate immune signaling and promote inflammatory cell recruitment [13,42].

The concurrent alterations in endothelial cell and vessel scores are also noteworthy, as endothelial dysfunction is a recognized feature of DCM that contributes to microvascular rarefaction and impaired myocardial perfusion [6], and ferroptosis in endothelial cells has been reported to exacerbate vascular dysfunction in diabetic settings [13]. Notably, in silico *Txnip* knockout in DCM cardiomyocytes prominently perturbed vascular signaling genes including *Vegfa* and *Adcy6* (Figure 7E), indicating that TXNIP-related cardiomyocyte network perturbation may be linked to vascular signaling programs in DCM myocardium.

TXNIP has been independently shown to facilitate NLRP3 inflammasome activation in cardiovascular tissues. For example, smooth muscle cell-specific *Txnip* deletion attenuates vascular calcification by suppressing NLRP3 activity and mitochondrial ROS production in vascular smooth muscle [43]. While these observations raise the possibility of a ferroptosis–inflammation–vascular remodeling axis in DCM, direct evidence for such a regulatory circuit in the diabetic myocardium is currently lacking. Future studies incorporating cell-type-specific functional approaches will be needed to determine whether the immune and vascular alterations observed here are mechanistically linked to hub gene-mediated ferroptotic signaling.

### 4.4. Translational Considerations

The identification of *Acot1* and *Txnip* as ferroptosis-related molecular signatures in DCM may have translational relevance. Both genes demonstrated reproducible classification performance across independent datasets and platforms, supporting their utility as tissue-level molecular signatures for pathological characterization of DCM. It should be noted, however, that these genes were identified in myocardial tissue and their current role is limited to tissue-level characterization rather than circulating biomarker applications.

From a therapeutic perspective, the distinct biological profiles of ACOT1 and TXNIP suggest complementary directions for future investigation. If the protective role of ACOT1 suggested by prior functional studies [34,35] is confirmed in DCM-specific models, strategies to enhance its expression may merit exploration. Potential approaches include viral-mediated gene delivery and the identification of upstream transcriptional activators. Conversely, several small-molecule TXNIP inhibitors, including verapamil and SRI-37330, have demonstrated efficacy in reducing *Txnip* expression in preclinical diabetic models [38], and ISRIB has shown promise in suppressing the ATF4/DDIT4/TXNIP ferroptosis cascade in septic cardiomyopathy [41]. Whether these pharmacological strategies can attenuate ferroptotic cardiomyocyte injury in the specific context of DCM remains to be determined. However, these translational considerations remain contingent on targeted gain- and loss-of-function studies that establish causal, rather than associative, roles for both genes in DCM-related ferroptosis.

### 4.5. Limitations

Several limitations should be acknowledged. First, all bioinformatics analyses were performed on murine transcriptomic datasets. Although rodent DCM models recapitulate key features of the human disease, direct clinical translation requires validation in human cardiac tissues. Furthermore, the small sample sizes inherent to publicly available GEO datasets may limit the statistical power and generalizability of the machine learning-derived signatures.

Additionally, while significant associations were identified between hub gene expression and immune cell infiltration scores, these correlations do not establish causality and require functional validation. Moreover, the single-cell RNA sequencing analysis was based on a single biological replicate per condition and should be interpreted as exploratory characterization of cell-type-specific expression patterns rather than definitive quantification. Finally, the experimental validation confirmed expression trends of ACOT1 and TXNIP but did not establish their causal roles in ferroptosis regulation, requiring targeted gain- and loss-of-function experiments.

## 5. Conclusions

This study employed an integrative bioinformatics and machine learning framework to identify *Acot1* and *Txnip* as ferroptosis-related molecular signatures in diabetic cardiomyopathy. Both prioritized candidate genes demonstrated strong classification performance in the discovery cohort and maintained discriminatory capacity in an independent external validation cohort using a different sequencing platform. Single-cell RNA sequencing provided exploratory evidence of cell-type-specific expression patterns, with *Txnip* broadly expressed across cardiac cell types and *Acot1* enriched in cardiomyocytes and endothelial cells. Spatial transcriptomic analysis supported disease-specific spatial association between candidate gene expression and ferroptosis driver scores in DCM myocardium. Experimental validation in HFD/STZ-induced DCM rats confirmed the upregulation of both genes at mRNA and protein levels. Collectively, these multi-layered findings support the involvement of *Acot1* and *Txnip* in ferroptosis-associated transcriptomic alterations in DCM and identify them as candidate targets for further mechanistic and therapeutic investigation.

## Data Availability

All public datasets analyzed in this study are available from the GEO (https://www.ncbi.nlm.nih.gov/geo/ accessed on 4 March 2026) under accession numbers GSE123975, GSE155377, GSE210611, GSE161931, GSE274500, GSE290095, and GSE290094.

## Supplementary Materials

The following supporting information can be downloaded at: https://www.mdpi.com/article/10.3390/cimb48090923/s1.
