# Supplementary material for: Ferroptosis Signatures in Diabetic Cardiomyopathy: Multi-Omics Discovery and Validation of ACOT1 and TXNIP
Source: Curr Issues Mol Biol. 2026 Sep 9;48(9):923. doi: 10.3390/cimb48090923 (PMC13605265; doi:10.3390/cimb48090923)
Supplement: Supplementary file 1 [file cimb-48-00923-s001.zip › FigureS1.pdf]

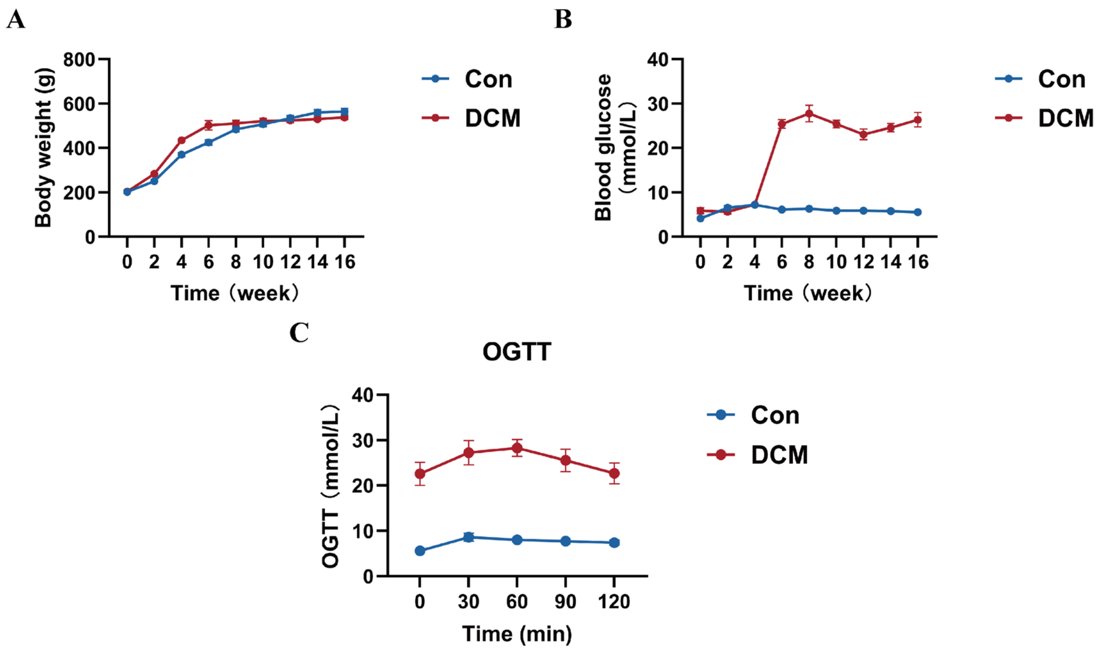

Figure S1. Metabolic phenotyping of the HFD/STZ-induced DCM rat model. (A) Body weight measured every two weeks throughout the observation period. (B) Random blood glucose monitored every two weeks. (C) Oral glucose tolerance test performed at study endpoint after an 8-h fast, with tail-vein blood glucose measured at 0, 30, 60, 90 and 120 min following oral gavage of glucose (2 g/kg body weight). n = 6 per group; data are presented as mean ± SEM. Con, control; DCM, diabetic cardiomyopathy; HFD, high-fat diet; STZ, streptozotocin; OGTT, oral glucose tolerance test.
