# Supplementary material for: Ferroptosis Signatures in Diabetic Cardiomyopathy: Multi-Omics Discovery and Validation of ACOT1 and TXNIP
Source: Curr Issues Mol Biol. 2026 Sep 9;48(9):923. doi: 10.3390/cimb48090923 (PMC13605265; doi:10.3390/cimb48090923)
Supplement: Supplementary file 1 [file cimb-48-00923-s001.zip › TableS1_cimb-4470051.pdf]

Supplementary Table S1

Summary of key statistical measures | Manuscript cimb-4470051

Table S1. Summary of key statistical measures. (A) Classification performance of the two hub genes in the discovery and external validation cohorts. (B) Sensitivity analysis of the six ferroptosis-related differentially expressed genes (FRDEGs) under Benjamini-Hochberg (BH) adjustment. (C) Hypergeometric testing of gene-set overlaps. (D) Cell-type abundance comparison between DCM and Con groups. (E) Spearman correlations between hub gene expression and cell-type abundance scores. (F) Spot-level spatial correlations between hub gene expression and ferroptosis driver scores. All multiple-testing correction used the BH method.

(A) Classification performance of hub genes

Gene	Cohort	n	AUC	95% CI	Expression *p*
Acot1	Discovery (GSE123975 + GSE155377 + GSE210611)	13 DCM / 13 Con	1.000	1.000–1.000	1.9×10^{-7}
Txnip	Discovery (GSE123975 + GSE155377 + GSE210611)	13 DCM / 13 Con	0.988	0.960–1.000	7.7×10^{-7}
Acot1	External validation (GSE161931 + GSE274500)	9 DCM / 9 Con	0.951	0.860–1.000	4.9×10^{-4}
Txnip	External validation (GSE161931 + GSE274500)	9 DCM / 9 Con	0.988	0.953–1.000	8.2×10^{-5}

AUC, area under the receiver operating characteristic curve. 95% CI computed by the DeLong method. Discovery-cohort values are in-sample estimates, as both genes were selected using this dataset. Expression *P* values from Wilcoxon rank-sum test.

(B) FDR sensitivity analysis of the six FRDEGs

Gene	$\log_2$FC	Nominal *p*	BH-adjusted *p*	Significant at BH $p < 0.05$
Acot1	+2.064	9.44×10^{-15}	1.71×10^{-10}	Yes
Pdk4	+1.689	6.10×10^{-12}	2.77×10^{-8}	Yes
Txnip	+1.045	5.74×10^{-9}	6.25×10^{-6}	Yes
Decr1	+0.531	6.70×10^{-9}	6.39×10^{-6}	Yes
Plin2	+0.668	1.10×10^{-7}	5.84×10^{-5}	Yes
Timp1	+0.832	7.88×10^{-4}	2.29×10^{-2}	Yes

The nominal threshold ($P < 0.05$, $|\log_2\text{FC}| > 0.5$) yielded 317 DEGs; the BH-adjusted threshold (adjusted $P < 0.05$, same fold-change criterion) yielded 261 DEGs. All six FRDEGs remained significant under BH adjustment.

(C) Hypergeometric testing of gene-set overlaps

Overlap	Observed	Expected	Fold enrichment	*p* value
DEG ∩ WGCNA module genes	176	38.6	4.57	$< 10^{-50}$
WGCNA module genes ∩ FerrDb	32	22.6	1.41	0.026
(DEG ∩ WGCNA) ∩ FerrDb (three-way)	6	3.6	1.66	0.153

Background: the WGCNA input gene set (top 50% most variable genes, N = 9071), which defines the universe of genes eligible for module membership. Set sizes: 317 DEGs, 1,103 WGCNA module genes (grey60 + magenta), 564 FerrDb ferroptosis regulators, of which 186 were present in the background gene set and were used for the enrichment calculation. The three-way intersection did not reach significance, indicating that this step functions as a biologically directed candidate filter rather than a statistically enriched selection; global enrichment of ferroptosis-related programmes is supported instead by gene-set GSEA ($P = 2.829 \times 10^{-7}$; Figure 2E).

(D) Cell-type abundance: DCM versus Con

Cell type	Con (mean)	DCM (mean)	Direction	Nominal *p*	BH-adjusted *p*	Significant at BH *p* < 0.05
Vessels	8.620	8.399	Lower in DCM	0.0025	0.0372	Yes
Eosinophils	8.864	8.415	Lower in DCM	0.0077	0.0446	Yes
Endothelial cells	8.010	7.707	Lower in DCM	0.0089	0.0446	Yes
CD8 T cells	5.091	4.855	Lower in DCM	0.0812	0.3046	No
Lymphatics	6.523	6.646	Higher in DCM	0.1824	0.5473	No
Neutrophils	4.177	4.278	Higher in DCM	0.3051	0.6904	No
Fibroblasts	7.237	7.266	Higher in DCM	0.3833	0.6904	No
Mast cells	4.130	4.227	Higher in DCM	0.4728	0.6904	No
T cells	5.363	5.447	Higher in DCM	0.5383	0.6904	No
NK cells	3.710	3.618	Lower in DCM	0.5383	0.6904	No
Granulocytes	4.902	5.023	Higher in DCM	0.5383	0.6904	No
Monocytes	4.578	4.662	Higher in DCM	0.5727	0.6904	No
Memory B cells	6.395	6.458	Higher in DCM	0.6444	0.6904	No
Monocytes / macrophages	5.567	5.533	Lower in DCM	0.6444	0.6904	No
B derived	4.915	4.881	Lower in DCM	0.9183	0.9183	No

Cell-type abundance scores estimated by mMCPcounter across the 26 discovery-cohort samples (13 DCM, 13 Con). Group comparisons by Wilcoxon rank-sum test, with BH adjustment applied across all 15 cell types. Basophils, the sixteenth mMCPcounter population, were excluded because estimated scores were zero in every sample. Three cell populations differed significantly after adjustment — vessels, eosinophils and endothelial cells — all lower in DCM; no comparison was significant at the nominal level only.

(E) Hub gene–cell type correlations

Gene	Cell type	Spearman rho	Nominal *p*	BH-adjusted *p*	Significant at BH *p* < 0.05
Acot1	Vessels	−0.561	0.0029	0.0430	Yes
Acot1	Endothelial cells	−0.510	0.0077	0.0528	No (nominal only)
Acot1	Eosinophils	−0.493	0.0106	0.0528	No (nominal only)
Acot1	Neutrophils	+0.308	0.1258	0.4717	No
Acot1	Monocytes	+0.237	0.2439	0.7014	No
Acot1	CD8 T cells	−0.220	0.2806	0.7014	No
Acot1	Granulocytes	+0.179	0.3803	0.8021	No
Acot1	Fibroblasts	+0.145	0.4788	0.8021	No
Acot1	Monocytes / macrophages	+0.134	0.5129	0.8021	No
Acot1	Mast cells	+0.128	0.5347	0.8021	No
Acot1	Memory B cells	+0.064	0.7564	0.8844	No
Acot1	T cells	+0.061	0.7665	0.8844	No
Acot1	Lymphatics	+0.061	0.7665	0.8844	No
Acot1	B derived	−0.045	0.8280	0.8872	No
Acot1	NK cells	+0.006	0.9775	0.9775	No
Txnip	Eosinophils	−0.586	0.0016	0.0247	Yes
Txnip	Vessels	−0.546	0.0039	0.0293	Yes
Txnip	Endothelial cells	−0.486	0.0117	0.0586	No (nominal only)
Txnip	Neutrophils	+0.427	0.0296	0.1109	No (nominal only)
Txnip	Mast cells	+0.357	0.0732	0.2195	No
Txnip	Monocytes	+0.338	0.0911	0.2278	No
Txnip	Lymphatics	+0.290	0.1513	0.3243	No
Txnip	Granulocytes	+0.259	0.2005	0.3759	No
Txnip	Memory B cells	+0.201	0.3239	0.5011	No
Txnip	NK cells	−0.197	0.3341	0.5011	No
Txnip	CD8 T cells	−0.169	0.4085	0.5571	No
Txnip	Monocytes / macrophages	+0.117	0.5706	0.7133	No
Txnip	B derived	+0.100	0.6263	0.7227	No
Txnip	Fibroblasts	+0.008	0.9696	0.9802	No
Txnip	T cells	+0.005	0.9802	0.9802	No

Spearman correlations computed across all 26 discovery-cohort samples. BH adjustment applied across the same 15 cell types separately for each hub gene. Three correlations remained significant after adjustment: Acot1–vessels, Txnip–eosinophils and Txnip–vessels. Negative rho values reflect higher hub gene expression alongside lower estimated cell-type scores in DCM samples, so these pooled correlations largely reflect disease-group differences rather than within-group gene–cell relationships.

(F) Spot-level spatial correlations with ferroptosis driver scores

Gene	Tissue group	Spearman rho	*p* value
Acot1	DCM	0.123	1.46×10^{-19}
Txnip	DCM	0.154	5.70×10^{-30}
Acot1	Con	< 0.05	—
Txnip	Con	< 0.05	—

Spatial transcriptomic data from GSE290094 (3 DCM, 4 Con). Ferroptosis driver scores computed by AddModuleScore. Correlations were calculated at the level of individual Visium spots and stratified by disease group. Control-tissue correlations were negligible in magnitude and are reported descriptively. The absolute rho values are modest, consistent with signal dilution across heterogeneous spots; the *P* values reflect the large number of spots rather than a strong effect size.
